# Treatment of pancreatic cancer in a nude mouse model using high-intensity focused ultrasound

**DOI:** 10.3892/etm.2012.744

**Published:** 2012-10-10

**Authors:** LIXIN JIANG, BING HU, QIAN GUO, LI CHEN

**Affiliations:** Department of Ultrasonography, Shanghai Jiaotong University Affiliated No. 6 Hospital, Shanghai 200233, P.R. China

**Keywords:** athymic nude mice, HIFU, pancreatic cancer

## Abstract

The purpose of this study was to determine whether high-intensity focused ultrasound (HIFU) is an effective treatment for pancreatic cancer in an athymic nude mouse model. Female athymic nude mice (n=44) were inoculated subcutaneously with 5–7×10^6^ SW1990 human pancreatic cancer cells. Thirty-six animals developed tumours and were randomly divided into three groups: HIFU (n=18), sham treatment (n=9) and control (n=9). The sonographic appearance of the tumours during therapy was recorded. After therapy, the tumours were examined transcutaneously by ultrasound every 4 days for 4 weeks. Tissue samples were taken from the treatment sites and histopathologically examined by light or electron microscopy. Two weeks after HIFU treatment, a 100% reduction in tumour volume was observed in all animals in the HIFU group, whereas tumours in the sham-treated and control groups continued to grow. Pathological examination of HIFU-treated tumour tissue samples showed complete coagulation necrosis in the tumour. Our results indicate that HIFU appears to be an effective therapy for pancreatic tumours in an athymic nude mouse model. Thus, the treatment may hold promise for the clinical treatment of late-stage and recurrent pancreatic cancer.

## Introduction

Pancreatic cancer is the fourth leading cause of cancer-related death in the United States and other Western countries (1). Due to the frequent delay in diagnosis, approximately 80% of patients have unresectable disease at presentation (2). Therefore, patients with locally advanced pancreatic cancer predominate in clinical practice. However, no effective modality has been identified thus far for the treatment of patients with locally advanced pancreatic cancer, although several studies have shown that chemoradiation offers a limited survival benefit (3,4). The median survival time is 6–10 months for patients with locally advanced pancreatic cancer and 3–6 months for those with metastatic disease. Due to the poor prognosis associated with late-stage and recurrent pancreatic cancer, new treatment options are required.

HIFU appears to be a candidate for the treatment of pancreatic cancer, where it may play a palliative role alongside chemotherapy. During HIFU treatment, focused ultrasound waves are emitted from a therapeutic transducer and absorbed by the target area, thereby inducing coagulation necrosis without causing damage to tissue in the path of the ultrasound beam (5,6). There have been few reports on the experimental use of HIFU in pancreatic cancer (7,8). In an effort to address this, we present the results of a study investigating whether HIFU is an effective treatment for pancreatic cancer in an athymic nude mouse model.

## Materials and methods

### Animals

Athymic nude mice (n=44), weighing 20–25 g, were supplied by the Shanghai Tumour Research Institute. The animals were inoculated subcutaneously with 5–7×10^6^ SW1990 cells. Cell line SW-1990 was established in 1978 from a pancreatic adenocarcinoma of a 56-year-old Caucasian male. It was derived from a pancreatic adenocarcinoma of ductal origin. Growing tumours exhibited characteristics of a Grade II adenocarcinoma similar to the original neoplasm (9). The animals were monitored every 4 days for the presence and size of tumours. Tumour volume was measured by transcutaneous ultrasound, and the long and short dimensions of the tumour were measured transcutaneously using a vernier calliper. As nude mice have thin skin, no hair and little subcutaneous fat, no correction for skin thickness was made (10,11). Tumour volume was calculated based on the assumption that each tumour was a regular ellipsoid.

Animals that developed tumours were randomised into three groups: HIFU treatment, sham treatment (scanned by ultrasound only, but received no HIFU) and control (no treatment). In the HIFU and sham groups, treatment was administered when the average tumour volume was approximately 0.1 cm^3^ (approximately 0.6 cm in diameter) after inoculation of SW1990 cells.

In the control group, tumours were allowed to grow to a maximum of 10% of the animal’s total body weight before the animals were euthanised.

This study received ethical and scientific approval from the ethics board of the Affiliated No.6 Hospital, Shanghai Jiaotong University, and complied with the Practice for Laboratory Animals guidelines in China.

### Diagnostic and therapeutic ultrasound

Diagnostic ultrasound was carried out using an Esaote MPX machine (Esaote S.p.A., Genova, Italy). Ultrasound images of the tumour prior and subsequent to treatment were recorded using a high-frequency probe whose frequency was 12.6 MHz.

HY2900 HIFU tumour therapy system (Wuxi Haiying Techonology, Wuxi, China) was used in this study. This device, which was designed and manufactured for clinical tumour treatments, comprised an ultrasonic diagnostic unit under the control of a central processing unit. The therapeutic transducer was a self-focused 6-element transducer with a diameter of 25 cm, and a focal length of 140 mm was fixed on the top of a water capsule filled with degassed water. A diagnostic transducer was localized in the center of the therapeutic transducer. The frequency of the diagnostic transducer was set at 3.5 MHz. Thus, the tissues in the path of therapeutic ultrasound waves could be viewed in diagnostic ultrasonic images. Ultrasonography was used to guide HIFU radiation and monitor therapeutic effects in real time. The maximum electrical power from the amplifier was 1.02 kW. The spatially averaged intensity level (I_SAL_) at −6dB was determined to be 9366 W/cm^2^ based on radiation force measurements and acoustic field mapping, producing a maximum acoustic power of 479.2 W. The water bag had an acoustic transparent membrane bottom to allow HIFU to transmit without obstruction, and ultrasound coupling gel was applied to eliminate air pockets trapped between the membrane and the skin of the nude mice. The frequency of the therapeutic ultrasound wave was 1.5 MHz. The focal region of the therapeutic transducers was an ellipsoid with dimensions of 8 mm along the beam axis and 1.15 mm in the transverse direction, which was calibrated using a PVDF needle hydrophone with a spot diameter of 0.5 mm in a tank filled with degassed water.

### Tumour tissue ablation

The animals were anaesthetised by intravenous injection of ketamine (2 ml/kg). After anaesthesia, the animals were maintained in the lateral decubitus position to allow the tumour to be viewed clearly with ultrasonography. The surface of the skin was kept in tight contact with the water tank. Sample images were taken under the guidance of B-mode ultrasound. The hypoechoic tumour tissue was treated point-by-point: therapy depth was 6 mm, and, as the tumour was small, treatment was performed in a horizontal mode with one layer. In all cases, pulses were applied at I_SAL_ = 589 W/cm^2^ and a pulse duration of 500 msec, with an exposure separation of 5 sec between each treatment point. The interval distance between treatment points was 1 mm. The sample images of each tumour were taken under the guidance of B-mode ultrasound immediately after therapy in the same manner as pretherapeutic imaging. If a hypoechoic region was observed after treatment, treatment was repeated until the region was hyperechoic. The sham-treated tumours were scanned by ultrasound but received no HIFU.

### Ultrasonic examination

After therapy, the tumours were examined transcutaneously by ultrasound every 4 days for 4 weeks. The examinations included two-dimensional ultra-sound, colour Doppler, and power Doppler. Examination was carried out using the same machine parameters each time. The tumour volume was measured by vernier calliper every 4 days for 4 weeks. The volume was calculated according to the following formula: V= 4/3 × π × ab2 = 4/3 × π × 1/2A × (1/2B)2 = 1/2AB^2^ (where V is volume and A and B the long and short dimensions, respectively; with a and b representing the half of the long and short dimensions, respectively) of the tumour in centimetres (12). Measurement was carried out three times, and the average value was used for tumour volume calculation. Tumour volumes were presented as the mean ± standard deviation of the mean (SD).

### Pathology

All 44 animals were sacrificed approximately 4 weeks after treatment, or 6 weeks after inoculation. Animals were euthanised by placing them in a CO_2_ chamber for 15–20 min. Tissue samples were taken from the treatment sites in the HIFU-treated animals, from tumours in the sham-treated and control animals, and from the lungs, livers and kidneys of all animals. All samples were fixed in 10% formalin, embedded in paraffin, and stained with haematoxylin and eosin for histopathologic examination by light microscopy and viewed at ×200 magnification. For electron microscopy examinations, samples were fixed in glutaric dialdehyde.

### Statistical analysis

Data were processed by the statistics software SPSS 12.0. One-way ANOVA analysis was employed. The difference was significant if the P-value was <0.05.

## Results

### Tumour volume and treatment time

Of the 44 animals that were inoculated, 36 developed tumours and these animals were either HIFU-treated (n=18), sham-treated (n=9) or received no treatment (control, n=9). After a period of approximately 2 weeks tumour masses reached 0.1 cm^3^ in volume, at which point they were considered suitable, based on the spatial dimensions of the HIFU focus, for HIFU or sham treatment. In the HIFU- and sham-treated groups, the average treatment times were 38±16.1 and 40±12.6 sec, respectively (Table I).

### Effects of HIFU treatment

**Figure 1:**
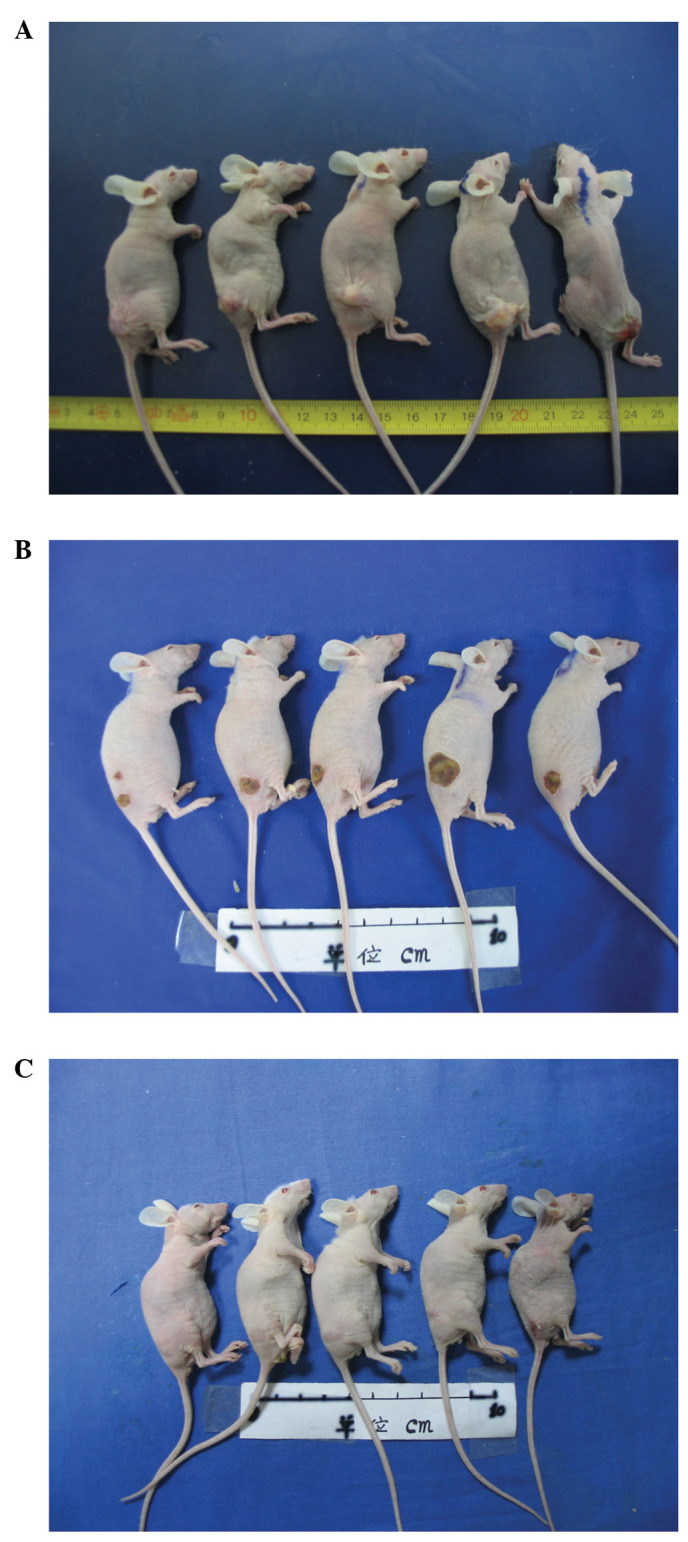
Photographs of nude mice with subcutaneous pancreatic cancer tumour in suprascapular region. (A) Untreated pancreatic cancer tumor prior to HIFU application with an approximate volume of 0.1 cm3. (B) Immediately after the application of HIFU, first- and second-degree skin burns were observed. (C) Four weeks after HIFU application, mice exhibited complete disappearance of the tumour. The skin repaired itself without any intervention.

Fig. 1 shows representative mouse tumours prior and subsequent to HIFU treatment. The subcutaneous tumour masses are evident prior to treatment (Fig. 1A). After the application of HIFU, first- or second-degree skin burns were observed (Fig. 1B). Four weeks after HIFU no tumour mass was apparent, and the treatment site appeared as a minor scar (Fig. 1C).

### HIFU treatment to decreased tumour volume

**Figure 2:**
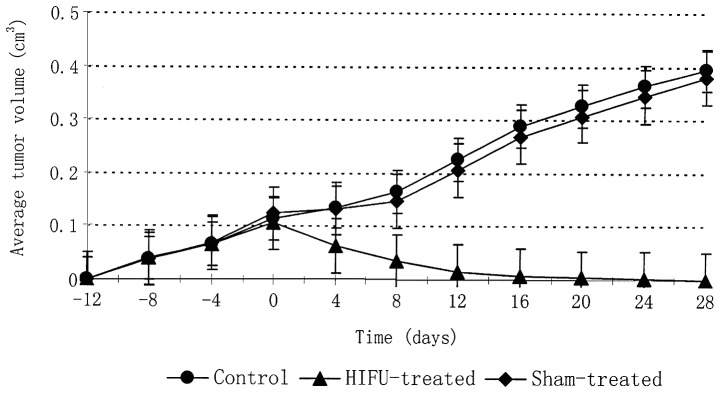
The average tumour volumes for HIFU-treated, sham-treated, and control animals were plotted as a function of time in days. The data were normalised in order for HIFU or sham treatment to be applied at day 0. The tumours had similar growth rates prior to HIFU or sham treatment. Following HIFU, a 100% reduction in volume was observed in all tumours within 28 days. By contrast, tumours of the sham-treated and control groups continued to grow, enlarging to approximately 400% of the tumour volume at the time of treatment.

The average ± SD of tumour volumes at the time of treatment was 0.12±0.06 and 0.10±0.12 cm^3^ for the sham and HIFU treatment groups respectively (Table I). The average tumour volumes for HIFU-treated, sham-treated, and control animals were plotted as a function of time in days (Fig. 2). The data were normalised in order for HIFU or sham treatment to be applied at day 0. All tumours had similar growth rates prior to HIFU or sham treatment. Following HIFU a 100% reduction in tumour volume was observed in all tumours within 28 days. By contrast, tumours of the sham-treated and control groups continued to grow, enlarging to approximately 400% of the tumour volume at the time of treatment. No metastases to liver, kidneys or lungs were observed in any of the groups.

### Echoicity following HIFU treatment

**Figure 3:**
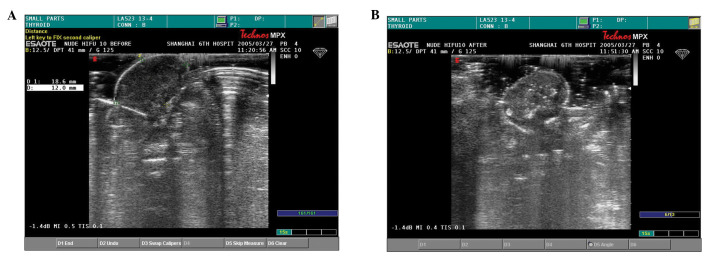
Ultrasound images of a tumor prior to and following HIFU treatment with an approximate volume of 0.1 cm^3^. (A) A tumor prior to HIFU treatment. Subcutaneous pancreatic cancer tumour appears as a hypoechoic area. (B) Treated tumour immediately after HIFU application. HIFU lesions appear as hyperechoic spots.

Before treatment, all tumours appeared hypoechoic under B-mode ultrasonic examination (Fig. 3A). Blood flow in and around the tumour, as detected by colour Doppler and power Doppler, was not abundant. In reconstructed three-dimensional images, the therapeutic region appeared hypoechoic in the horizontal plane. The echoicity of the therapeutic region increased during HIFU treatment, at the end of which a bright hyperechoic signal was evident (Fig. 3B). Tumours in the sham-treated and control groups remained hypoechoic.

### HIFU eradicated viable tumour cells

**Figure 4:**
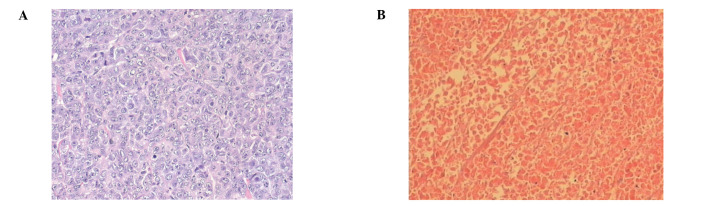
Light microscopy of pancreatic tumour prior to and following high-intensity focused ultrasound (HIFU). (A) In sham-treated and control animals, the nuclei of viable tumour cells are in a central configuration [haematoxylin and eosin (H&E) stain; original magnificaton, ×200]. (B) Four weeks after HIFU, the site showed coagulative necrosis and no remaining viable tumour cells (H&E; original magnification, ×200).

Under pathological examination, the tumours appeared as subcutaneous masses prior to treatment. First- or second-degree skin burns were observed in almost all of the HIFU-treated animals. In all cases, the skin repaired itself without any intervention. Light microscopic examination of tumour tissue taken 4 weeks after treatment revealed tumour cells that appeared viable and had centrally located nuclei in animals in the sham-treated and control groups (Fig. 4A), whereas no remaining viable tumour cells were observed in the HIFU-treated animals (Fig. 4B).

**Figure 5:**
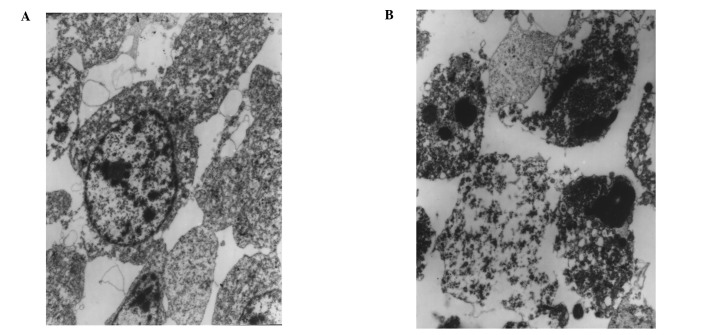
Electron microscopy of pancreatic tumour after high-intensity focused ultrasound (original magnification, ×5000). (A) Mitochondria and rough endoplasmic reticulum have degenerated. (B) Apoptotic bodies can also be observed.

Electron microscopy of tumour tissue taken after HIFU showed extensive disruption of cell membranes, nuclei and cytoplasmic components (Fig. 5A). In addition to karyorrhexis, cytoplasmic structures such as mitochondria and rough endoplasmic reticulum had disappeared, and chromatin was clumped at the periphery of nuclei; only some cellular ground substance remained. Apoptotic bodies were also observed (Fig. 5B).

## Discussion

We studied the efficacy of high-intensity focused ultrasound (HIFU) for the treatment of pancreatic cancer in a nude mouse model. After HIFU treatment, the tumour volumes in mice were significantly reduced with minimal side effects. Treatment using HIFU requires an accurate visualisation method for targeting and monitoring therapy. We found HIFU-treated tissue to have a characteristic appearance under ultrasound: in our study, a bright hyperechoic region appeared in the HIFU-treated region. The appearance of a persistent hyperechoic region following HIFU treatment has been demonstrated to be due to gas following boiling of tissue. Boiling, that is, the growth of vapour bubbles, occurs due to HIFU-induced temperature rise. In this sense, boiling is to be distinguished from cavitation, which occurs due to HIFU-induced pressure oscillations (13). Our results also indicate that B-mode ultrasound may provide a valuable real-time method for monitoring the HIFU treatment of these tumours.

After HIFU treatment some malignant cell islands in the centre of the lesion failed to show morphological changes characteristic of cell necrosis under light microscopy. However, electron microscopy confirmed the presence of ultrastructural cellular damage. Electron micrographs revealed complete disruption of the nuclei and cytoplasmic ultrastructure, indicating that the tumour cells in question had undergone necrosis after HIFU exposure. Although most initial cell death in tissues exposed to HIFU fields is caused by cell necrosis due to thermal injury, HIFU is also able to induce apoptosis. In apoptotic cells, the nucleus of the cell self-destructs, with rapid degradation of DNA by endonucleases. The primary mechanism of cell death by hyperthermia is apoptosis (14).

Two weeks after treatment, a 100% reduction in tumour volume was observed in all animals in the HIFU treatment group, whereas tumours in the sham-treated and control groups continued to grow. Similar findings were reported in previous studies (10,12).

We observed that the skin overlying the treatment area turned white immediately after HIFU application, indicating a skin burn. We considered the burn to be due to three causes, as follows: i) The difference in acoustic impedance between skin and either water or air, leading to ultrasound beam reflection and refraction, and the conversion of mechanical energy to heat at the skin surface. ii) Air bubbles in the barrier gel during treatment, which may have contributed to suboptimal coupling conditions. Future studies should ensure that degassed procedure is carried out. iii) The pancreatic tumours in our model were located subcutaneously, reducing the focus-to-skin distance during the HIFU tumour ablation process. In order to avoid skin injury, it would be necessary to ensure that the focus-to-skin range is at least 1 cm; if this is not possible, the HIFU duty factor and exposure time should be decreased. However, the relationship between therapeutic dose, therapeutic depth and skin burns has not been thoroughly investigated and requires further study (15).

Cell line SW-1990 was established from a pancreatic adenocarcinoma of a 56-year-old Caucasian male. It was derived from a pancreatic adenocarcinoma of ductal origin. Growing tumours exhibited characteristics of a Grade II adenocarcinoma similar to that of the original neoplasm. Two types of mouse model of pancreatic cancer have been used in several studies. One is the subcutaneous transplantation model and the other is the orthotopic transplantation model. In our study, HIFU was used to treat subcutaneously implanted tumours. This mouse model did not exhibit metastatic disease. However, if the tumour grew large enough, it would indicate metastatic disease in the lung and/or liver. The model did not simulate the situation and some clinical settings of human pancreatic cancer. Therefore, the use of the orthotopic transplantation mode should be investigated in future studies (9).

Limitations of this study also included the small number of samples and the relatively limited length of follow-up monitoring. These limitations are to be addressed in future studies. In the clinical setting, the application of HIFU for the treatment of pancreatic cancer is likely to be influenced by additional factors, such as the degree of penetration of the abdominal wall and the possibility of interference by abdominal gas. Further basic and clinical research is required before HIFU becomes a safe and effective clinical therapy for pancreatic cancer tumour. However, the results of this study indicate that HIFU holds great promise as an alternative noninvasive method of treatment for pancreatic cancer (16,17).

## Figures and Tables

**Table I:** Pre- and post-treatment tumour volume and treatment time.

Groups	n	Pre-treatment tumour volume (cm^3^)	Post-treatment tumour volume (cm^3^)	Treatment time (sec)
HIFU-treated	18	0.10±0.12	0	38±16.1
Sham-treated	9	0.12±0.06	0.42±0.39	40±12.6
Control	9	0.11±0.06	0.39±0.36	NA
No tumour	8	NA	NA	NA

Total n=44. Data are expressed as average ± SD. HIFU, high-intensity focused ultrasound; NA, not applicable.
